# Cell Wall Composition Impacts Structural Characteristics of the Stems and Thereby the Biomass Yield

**DOI:** 10.1021/acs.jafc.1c06986

**Published:** 2022-03-02

**Authors:** López-Malvar Ana, Santiago Rogelio, Souto Xose Carlos, Malvar Rosa Ana

**Affiliations:** † Facultad de Biología, Departamento de Biología Vegetal y Ciencias del Suelo, Universidade de Vigo, As Lagoas Marcosende, 36310 Vigo, Spain; ‡ Agrobiología Ambiental, Calidad de Suelos y Plantas (UVIGO), Unidad Asociada a la MBG (CSIC), 36310 Vigo, Spain; § Misión Biológica de Galicia (CSIC), Pazo de Salcedo, Carballeira 8, 36143 Pontevedra, Spain; ∥ Departamente Ingeniería Recursos Naturales Y Medio Ambiente, E.E. Forestales, Universidade de Vigo, 36005 Pontevedra, Spain

**Keywords:** *Zea mays*, cell wall, stem characteristics, biomass, maize, plant architecture

## Abstract

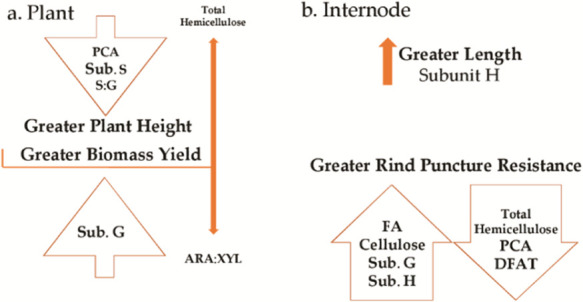

Maize stalks support leaves and reproductive structures and functionally support water and nutrient transport; besides, their anatomical and biochemical characteristics have been described as a plant defense against stress, also impacting economically important applications. In this study, we evaluated agronomical and stem description traits in a subset of maize inbred lines that showed variability for cell wall composition in the internodes. Overall, a great proportion of lignin subunit G and a low concentration of *p*-coumaric acid and lignin subunit S are beneficial for greater rind puncture resistance and taller plants, with a greater biomass yield. Also, the greater the proportions of subunit H, the longer the internode. Finally, the lower the total hemicellulose content, the greater the rind puncture resistance. Our results confirmed the effect of the cell wall on agronomic and stalk traits, which would be useful in applied breeding programs focused on biomass yield improvement.

## Introduction

The structure and function of the plant cell wall are controlled by how each of its components interacts within the cell wall. This strong assembly, apart from providing structural support and rigidity to the cell and determining its size and shape, also provides resistance to abiotic and biotic stresses and communication among cells.^1,2^ Furthermore, the framework constituted by the cell wall is closely related to the growth and fitness of the plant and is expected to determine the functional characteristics of the stem, which are closely related to yield.^3,4^

From a breeder point of view, the first goal of the crop improvement is, on the one hand, to obtain increased grain yield, considered as the potential of the grain production and increased biomass yield expressed as tons of biomass produced per hectare.^5,6^ In maize, increases in maize grain have been accompanied by increases in biomass, which indicates that breeding for biomass yield would not compromise grain yield.^7^ Furthermore, increases in biomass or stover yield have been also a target trait for biofuel production and forage digestibility.^8,9^

Because cell walls constitute more than 50% of the dry biomass weight, improvement of biomass relies largely on the cell wall components and anatomical arrangement of the stems and also conditioning the plant height.^9−12^ However, increases in plant height must have to deal with stem lodging losses. Stem lodging, caused by the bending or breaking of the stalk, is greatly impacted by the stalk strength and stem morphological traits; therefore, it could be said that maize stem strength impacts both grain yield and silage quality.^10,13,14^

Research on cell wall composition and its influence on basic and applied aspects of maize stem strength would be important steps in maize breeding and improvement.^10,15^ Overall, in the current study, we evaluated agronomical and stem description traits in a subset of maize inbred lines that showed variability for cell wall composition, the main goal being to identify cell wall components that can be used in applied breeding programs.

## Materials and Methods

### Plant Material and Experimental Design

A set of 20 inbred lines was tested through two consecutive years (2016 and 2017) in Pontevedra (Spain, 42° 24′ 22.3″ N, 8° 38′ 28.16″ W, 20 m above sea level). The set of inbred lines evaluated can be subdivided into three subsets: (i) inbreed lines included in previous evaluations for resistance to *Sesamia nonagrioides* or *Ostrinia nubilalis*, (ii) inbreds used in hybrid combinations for bioenergy and for silage, and (iii) inbreds that perform well in hybrid combinations. A complete and detailed description of the inbreds evaluated can be found in ref (16).

In both trials, the set was evaluated following a random block design with three repetitions. In 2017, the set was reduced to 19 because there was not enough stock for the inbred line PB130. The experimental plots consisted of three rows, with 15 double-kernel hills each, with a total surface of 0.14 m^2^ per plot, and with a final density of ∼70,000 plants ha^–1^ after thinning. The trials were maintained with local agronomical practices.

### Agronomic Traits

#### Biomass Yield

Seventy days after silking, considering it as days from planting until half of the plants in the plot showed visible silks, the plots were harvested. Two to ten plants without ears from each plot were collected, weighed, and chopped from which a stover sample was collected (sample fresh weight) for estimating the percentage of stover dry matter. For that, the fresh stover was pre-dried (35 °C) in a forced air-drying chamber, dried on a stove (60 °C), and again weighed after a week (sample dry weight).

Determination of biomass yield in mg ha^–1^ was done as follows
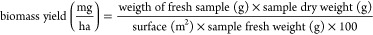


The surface was calculated as the number of plants per plot multiplied by the space between rows (0.80 m) and the space between plants (0.18 m). Following this equation, the biomass yield corresponds to the maximum yield.

#### Stem Lodging

Calculated at harvest, the sum of broken plants (split underneath the main ear) divided by the total number of plants in the plot was calculated. Stem lodging is expressed in percentage.

### Stem Description Traits

Rind puncture resistance, the total number of internodes, and internode diameter were recorded 55 days after flowering, and the rest of the stem description traits were studied 70 days after flowering. A more detailed description of the methodology can be found in López-Malvar et al.^16^

Briefly, plant height was calculated as the mean of plant height (in cm) measured from the base of the plant until the flag leaf of five plants per plot; internode length was calculated as the total number of internodes divided by the height of the plant; in five plants, rind puncture resistance was measured from the maximum force required to puncture the rind (in kg/section) on one side of the stalk using an Accuforce Cadet Force Gauge (Ametek, Mansfield and Green Division, Largo, FL); from the same five internodes, using an electronic caliper, the diameter was recorded in millimeters.

### Biochemical Traits

The complete characterization of the cell wall was performed in the second internode below the main ear from five plants per plot, collected 55 days after silking. The complete description of the methodology can be found in López-Malvar et al.^16^

Briefly, cellulose was quantified in crude cell walls by the Updegraff method;^18,19^ the hemicellulose composition was determined using a high-performance anion exchange chromatograph (Carbopac PA-10; Dionex, Camberley, Surrey, UK); as described previously by Jones et al. (2003),^20^ it included the quantification of glucose, galactose, fucose, arabinose, rhamnose, xylose, mannose, arabinose/xylose ratio, and glucuronic and galacturonic acid (the sum of all of them would be considered further on as the total hemicellulose content); the total lignin content was determined by the Klason Lignin protocol;^21^ the subunit composition was determined by thioacidolysis, followed by gas chromatography–mass spectrometry;^22^cell wall-bound hydroxycinnamate quantification was performed using high-performance liquid chromatography following the protocol described in Santiago and col.^23,24^

### Statistical Analysis

#### Contrast Analysis

The SAS mixed model procedure (PROC MIXED) of the SAS program (version 9.4)^25^ was used for the individual and combined analyses of variance for each trait. Using the combined data for the analysis across years, the best linear unbiased estimator (BLUE) for each inbred line was calculated. We considered as fixed effects inbred lines and as random effects years, replication within years, and lines × year. We used Fisher’s protected least significant difference for means comparison.

After that analysis, inbred lines were qualitatively classified, according to their BLUEs, in high, intermediate, and low groups for agronomic and stem traits (Table 1); high and low groups differing by *p* < 0.05. With the qualitative data set, mean comparisons for groups with contrasting values were performed to look for differences in cell wall composition.

**Table 1:** Inbred Lines under Study Were Qualitatively Classified According to the BLUEs for Biomass Yield and Stem Description Traits Evaluated in 2016 and 2017

inbred	plant height (cm)	internode length (cm)	internode diameter (mm)	rind runcture resistance (kg/section)	Stover yield (mg/ha)
A509	low	intermediate	intermediate	low	low
A632	high	low	low	intermediate	high
A654	low	low	intermediate	intermediate	intermediate
C103	high	high	intermediate	high	high
CO348	low	low	intermediate	high	intermediate
CO384	high	low	intermediate	intermediate	high
CO442	high	low	intermediate	low	high
CO444	intermediate	low	low	intermediate	intermediate
EC212	intermediate	low	intermediate	high	intermediate
EP105	high	intermediate	intermediate	intermediate	intermediate
EP125	high	high	intermediate	low	low
EP17	high	low	high	high	high
EP42	intermediate	low	low	intermediate	intermediate
EP47	high	high	high	intermediate	high
EP53	low	high	intermediate	high	low
EP86	high	intermediate	low	low	low
F473	low	low	low	intermediate	low
PB130	low	low	high	intermediate	intermediate
W182B	low	low	high	low	low
W64A	intermediate	low	high	intermediate	intermediate

### Multiple Linear Regression Analysis

For understanding the relationship between agronomic and stem description traits and cell wall components, we studied a multiple linear regression model using the BLUEs. For this analysis, we used, in SAS,^25^ the stepwise method following the PROC REG procedure. Variables with a significance value of less than 0.15 were not selected to take part in the regression model. We considered as dependent variables agronomic and stem description traits; as independent variables, we considered cell wall components.

## Results

Inbred lines differed significantly for biomass yield and stem description traits. There were no significant differences for stem lodging, so it was not included in the contrast analysis or in the multiple linear regression (Supporting Information Table S1).

### Contrast Analysis

Significant differences between high- and low-contrast groups for every trait are shown in Table 2. Values for non-significant traits in the contrast analysis are included in Supporting Information Table S2. Inbred lines presenting the greatest biomass yield showed a lower concentration of *p*-coumaric acid (PCA), low proportion of lignin subunits S and H, low S/G ratio, and, on the contrary, a greater proportion of subunit G.

**Table 2:** Contrast Analysis of Inbred Lines Attending to Contrasting Values of Biomass Yield and Agronomic Stem Description Traits^a^

	classification group			
cell wall component	high	intermediate	low	LSD
Biomass Yield (mg/ha)				
PCA (mg/g)	11.54	12.77	13.94	0.907
S subunit (%)	55.28	57.83	57.83	0.982
S/G ratio	1.317	1.44	1.479	0.052
G subunit (%)	42.17	39.27	39.27	0.851
H subunit (%)	2.548	1.95	2.910	0.052
Plant Height (cm)				
S subunit (%)	55.65	57.26	58.88	0.98
S/G ratio	1.34	1.42	1.52	0.052
G subunit (%)	41.64	40.75	38.85	0.89
H subunit (%)	2.72	2.28	2.28	0.38
Internode Length (cm)				
H subunit (%)	2.95	2.26	2.26	0.44
Rind Puncture Resistance (kg/section)				
PCA (mg/g)	11.38	12.70	13.90	0.99
DFA 8-5-l (mg/g)	0.046	0.060	0.062	0.008
DFA 8-5-b (mg/g)	0.088	0.105	0.105	0.156
DFA 5-5 (mg/g)	0.067	0.087	0.086	0.012
DFAT (mg/g)	0.274	0.338	0.326	0.045
cellulose (mg/g)	441.63	441.63	382.11	34.324
galactose (mg/g)	4.855	5.600	8.285	2.926
galacturonic acid (mg/g)	6.734	9.230	10.166	2.422
glucuronic acid (mg/g)	2.472	2.748	3.936	1.935
arabinose (mg/g)	8.002	9.218	12.248	3.016
mannose (mg/g)	2.429	2.586	3.699	0.80
xylose (mg/g)	20.87	24.21	24.75	3.051
H subunit (%)	2.248	2.356	2.703	0.398
G subunit (%)	41.15	40.73	39.77	1.041
S/G ratio	1.39	1.40	1.45	0.06

a Only cell wall components that significantly differ among groups are included. LSD: least square distance (*P* ≤ 0.05) DFA 8-5-l: Diferulic acid 8-5-linear; DFA 8-5: diferulic acid 8-5; DFA 8-5-b: diferulic acid 8-5-benzofuran; DFAT: total diferulic acids * some missing data for individual traits and inbreds could interfere in the final ratio calculations of the groups.

In the same way, but attending to the plant height, the taller plants presented lower proportions of a lignin subunit S and S/G ratio, and higher proportions of lignin subunit G; however, the H subunit showed the opposite trend for biomass yield.

Regarding the internode description traits, the greater proportion of subunit H, the longest the internode, in accordance with plant height results. Contrast groups for the internode diameter did not differ for any cell wall trait. Finally, inbred lines showing a greater resistance to puncture were the ones showing the greatest cellulose content and the greatest proportions of subunit G, the lowest concentrations of cell wall-bound hydroxycinnamates (namely, PCA and diferulates), lowest total hemicellulose content (galactose, glucuronic and galacturonic acid, arabinose, xylose, and mannose), and the lowest proportions of lignin subunit H and S/G ratio (Table 2).

### Multiple Linear Regression

We found that a greater proportion of the lignin subunit G and a greater total hemicellulose content (mainly galactose) increase the biomass yield; on the contrary, a greater galacturonic acid and arabinose/xylose ratio decreases the biomass yield (Table 3).

**Table 3:** Multiple Linear Regression Model (Using Stepwise Selection) of Biomass Yield and Stem Description Traits on Cell Wall Composition of a Set of Inbred Lines Evaluated in 2016 and 2017^a^

stepwise selection		
biomass yield (mg/ha)	*R*^2^ partial	*R*^2^
subunit G (%)	0.31	0.31
arabinose/xylose ratio	0.14	0.46
total hemicellulose (mg/g)	0.09	0.55
galacturonic acid (mg/g)	0.07	0.62
galactose (mg/g)	0.07	0.69
model	biomass yield: −16.11997 + 0.48330 × *G* – 5.68752 × ARA/XYL + 0.55854 × galactose – 0.38920 × galacturonic acid + 0.03730 × total hemicellulose	

stepwise selection		
plant height (cm)	*R*^2^ partial	*R*^2^
subunit S (%)	0.34	0.34
model	plant height = 469.3563 – 5.72250 × S	

stepwise selection		
rind puncture resistance (kg/section)	*R*^2^ partial	*R*^2^
galacturonic acid (mg/g)	0.36	0.36
arabinose/xylose Ratio (mg/g)	0.11	0.47
glucose (mg/g)	0.07	0.53
model	rind puncture resistance: 3.83618 + 0.01978 × glucose – 0.15986 × galacturonic acid – 0.58579 × arabinose/xylose ratio	

a *R*^2^: total % of the variance explained by the model; *R*^2^ partial: % of the variance explained by each trait.

We found that 34% of the variance for plant height was affected by the lignin subunit S, with a negative effect. In the case of the internode length and internode diameter, no variable met the 0.15 significance level to be included in the model (Table 3). Rind puncture resistance was mainly affected by the galacturonic acid concentration and arabinose/xylose ratio, negatively, and positively by glucose, reporting 53% of the variation for rind puncture resistance (Table 3).

## Discussion

Our results confirm that biomass, stem strength, and other stem features such as plant height or internode length rest on the organization and composition of the stem cell walls. Secondary cell wall formation, characterized by lignin deposition, seems to play a central role in maize stem characteristics.

### Contrast Analysis

In the contrast analysis, we noted that it is not the total lignin content but the lignin subunit composition which is the trait that most influences different groups of lines classified and in high and low groups for plant biomass and stem architecture; the lignin with higher proportions of subunit G detrimental to subunit S is valuable for increased biomass yield, plant height, and rind puncture resistance (Table 2). The composition and proportion of the subunits highly influence the molecular structure of lignin. It affects the degree of crosslinking with the polysaccharides and also the branching of the polymer, affecting, as it has been demonstrated, economically important processes such as biofuel production and digestibility.^16,26^ In addition, some other related phenylpropanoids also contributed to biomass yield and anatomical traits of the stems. We have shown that a great concentration of PCA in the cell wall is unfavorable for increasing biomass yield and rind puncture resistance. Most PCA is bound to S units in lignin, esterified to the γ-position of phenylpropanoid sidechains.^27^ The PCA acylation influences the bonding mode of S lignin units and the spatial organization of lignin and, by consequence, also the way that lignin and polysaccharides interact.^28^ In this sense, S-type lignin presents a more linear structure^29^ with almost no branching and with a lesser degree of polymerization; lignin G is more condensed than lignin S.^30^ In our case, S-type lignin is detrimental for increases in the biomass yield, plant height, and rind puncture resistance.

The network formed by the fibers within the cell wall (cellulose–lignin–hemicellulose) is believed to define the functional properties of the stems.^31^ We found that increases in cellulose would favor greater rind puncture resistance and, therefore, stalk strength, while greater concentrations of total hemicellulose content would be disadvantageous for rind puncture resistance. Increases in rind puncture resistance and formation of the cortex tissue have been closely related with cellulose and lignin deposition, serving as structural support to the cell wall.^32^ Moreover, cellulose compositional features, such as crystallinity, have been related to stalk lodging and stalk strength, which could be associated with rind puncture resistance, as previously mentioned.^15^ The positive association between the quantity of cellulose amorphous regions and the arabinose-substitution of xylans has been proved; also, the negative effect that increasing levels of arabinose have on cellulose crystallinity has been demonstrated.^33,34^ In the contrast analysis, the group of inbred lines presenting the higher rind puncture resistance present a reduced arabinose/xylose ratio and thus a reduction in the arabinose content. In a cell wall presenting a low concentration of arabinose, the hemicellulose and cellulose chains tend to interact through hydrogen bonds, which would contribute to more crystalline cellulose, which is more uniform, ordered, and hard; this could indicate a greater resistance to puncture. Contrary to cellulose, hemicelluloses are not chemically uniform. Xylan containing β-(1,4)-linked xylose residues is one of the most complex heteroxylans in the fiber of maize.^35^ Based on Appeldoorn et al.^36^ and Van Eylen et al.,^37^ a reduced incidence of uronic acid, acetic acid, and arabinose side groups in glucuronoarabinoxylans would drive changes in the properties of the cell wall. Contrast analysis showed that the presence of more glucuronic acid and galacturonic acid may contribute to a less strengthened stalk that is less resistant to puncture.

Finally, the mechanical resistance granted by DFAs would make us think that a cell wall with a greater strength and a higher tissue toughness would also present a greater resistance to the penetrometer, however, regarding our contrast analysis, the group of inbred lines showing the greatest rind puncture resistance showed the lowest concentrations of diferulates. However, our results are in accordance with the ones obtained by Manga-Robles et al.^15^ in a previous study. They observed a significantly higher level in diferulic individual dimers in inbred lines showing a low rind penetrometer strength. With respect to the plasticity of the cell wall, we may argue that some of the other components of the cell wall have a more significant part in the strengthening and support like, for this panel of inbred lines, great cellulose content or lignin presenting a low S/G ratio, which would increase the rind puncture resistance.

### Multiple Linear Regression

Mainly, the results obtained in the multiple linear regression analysis support the ones obtained in the contrast analysis. Again, the influence of lignin subunit composition and how PCA acetylation of the lignin subunit affected the final lignin structure showed significant effects on biomass yield and plant height. Lignin with a greater proportion of subunit G may be beneficial for greater biomass yield, and lignin presenting lower proportions of subunit S would produce taller plants.

We have already mentioned how the fiber proportion of the cell wall takes part in determining the stem anatomical characteristics; according to our results, the structural support granted by total hemicellulose content produces greater biomass yields.

Besides, we found that a reduced arabinose/xylose ratio and lower concentrations of galacturonic acid decrease both rind puncture resistance (in accordance with contrast analysis) and biomass yield. As previously explained for the contrast analysis results, the influence of matrix polysaccharides (total hemicellulose content) has been confirmed to affect the rind puncture resistance; and in the same way, it could affect the biomass yield. The negative relationship between the arabinose content and the cellulose crystallinity has been demonstrated. The intra- and intermolecular hydrogen bridges within the cellulose result in a crystalline configuration that gives cellulose mechanical solidity, which may be beneficial for biomass increases.^38^

**Figure 1:**
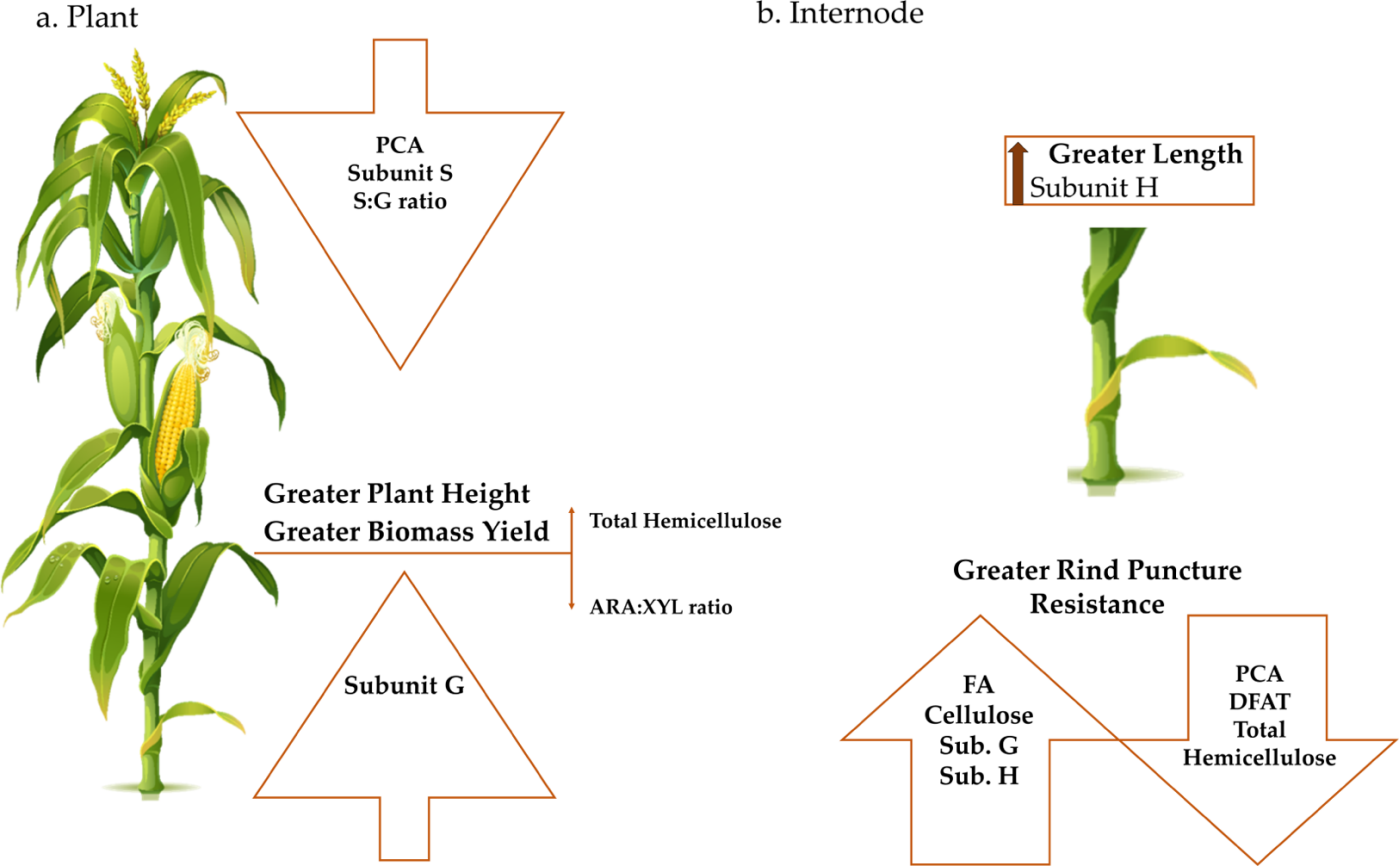
Graphical summary of the results obtained. (a) Results concerning the whole plant and (b) results concerning the second internode below the main ear.

In this representative material, S-type lignin accompanied by increases in PCA would be detrimental to the biomass yield, plant height, and rind puncture resistance, whereas cell walls richer in cellulose and with a lower proportion of total hemicellulose would be beneficial for stalk strength (Figure 1). These results prove that cell wall composition clearly influences the structural characteristics of the maize stems and thereby can be useful to improve maize biomass yield.

## Supporting Information Available

Means of 20 inbred lines evaluated for agronomic and stem description traits; contrast analysis of inbred lines attending to contrasting values of biomass yield and agronomic stem description traits; and means for cell wall components with non-significant differences among groups (PDF)

## Abbreviations

BLUEs: best linear unbiased estimators
PCA: *p*-coumaric acid
DFA 8-5-l: diferulic acid 8-5-linear
DFA 8-5: diferulic acid 8-5
DFA 8-5-b: diferulic acid 8-5-benzofuran
DFAT: total diferulic acids
G: subunit
