# Cell Wall Composition Impacts Structural Characteristics of the Stems and Thereby Biomass Yield

**DOI:** 10.1021/acs.jafc.2c04380

**Published:** 2022-06-29

**Authors:** Ana López-Malvar, Rogelio Santiago, Xose Carlos Souto, Jaime Barros-Rios, Leonardo D. Gómez, Rosa Ana Malvar

**Affiliations:** † Facultad de Biología, Departamento de Biología Vegetal y Ciencias del Suelo, Universidade de Vigo, As Lagoas Marcosende, 36310 Vigo, Spain; ‡ Agrobiología Ambiental, Calidad de Suelos y Plantas (Universidade de Vigo), Unidad Asociada a la Misión Biológica de Galicia (MBG), Consejo Superior de Investigaciones Científicas (CSIC), 36310 Vigo, Spain; § Misión Biológica de Galicia (MBG)Consejo Superior de Investigaciones Científicas (CSIC), Pazo de Salcedo, Carballeira 8, 36143 Pontevedra, Spain; ∥ Escola de Enxeñaría Forestal, Departamento de Ingeniería de los Recursos Naturales y Medio Ambiente, Universidade de Vigo, 36005 Pontevedra, Spain; ⊥ Division of Plant Sciences and Technology, College of Agriculture Food and Natural Resources and Interdisciplinary Plant Group, Christopher S. Bond Life Sciences Center, University of Missouri, Columbia, Missouri 65211, United States; # Centre for Novel Agricultural Products (CNAP), Department of Biology, University of York, Heslington, York YO10 5DD, United Kingdom

After a discussion with all of the authors, we have agreed on the following changes to the original manuscript:

The authors and affiliations have been corrected as presented in this Addition and Correction.

The acknowledgments have been corrected as follows: The authors thank Dr. Rachael Simister for the technical assistance of matrix polysaccharide analyses. The authors are grateful to Prof. Simon J. McQueen-Mason for the support at the Centre for Novel Agricultural Products (CNAP), University of York, York, U.K., and the Dixon laboratory at University of North Texas for support with the lignin compositional analyses.

The author contributions have been corrected as follows: Rosa Ana Malvar and Rogelio Santiago conceived of the study. Rogelio Santiago, Rosa Ana Malvar, and Ana López-Malvar participated in the experimental design, carried out the field trials, and participated in sample collection. Ana López-Malvar carried out biochemical determinations and statistical analysis. Ana López-Malvar wrote the draft. Jaime Barros-Rios and Leonardo D. Gómez assisted in biochemical analysis and Results and Discussion. Xose Carlos Souto contributed to the Results and Discussion. All authors read and approved the final manuscript.

