# Supplementary material for: Cell Wall Composition Impacts Structural Characteristics of the Stems and Thereby the Biomass Yield
Source: J Agric Food Chem. 2022 Mar 2;70(10):3136–41. doi: 10.1021/acs.jafc.1c06986 (PMC8931758; doi:10.1021/acs.jafc.1c06986)
Supplement: Supplementary file 1 — jf1c06986_si_001.pdf [file jf1c06986_si_001.pdf]

Cover Art Caption

Graphical summary of the results obtained. A: Results concerning the whole plant; b: results concerning the second internode below the main ear.

Supplementary Table 2. Contrast analysis of inbred lines attending to contrasting values of biomass yield and agronomic stem description traits. Means for cell wall components with **non-significant differences** among groups are included.

| | Classification Group | | | |
|---|---|---|---|---|
| **CW Component** | **High** | **Intermediate** | **Low** | **LSD** |
| **Biomass yield (Mg/ha)** | | | | |
| FA (mg/g) | 3.133 | 3.038 | 3.003 | 0.413 |
| DFA 8-5-l (mg/g) | 0.0589 | 0.053 | 0.061 | 0.008 |
| DFA 5-5 (mg/g) | 0.080 | 0.084 | 0.083 | 0.011 |
| DFA 8-O-4 (mg/g) | 0.084 | 0.079 | 0.074 | 0.016 |
| DFA 8-5-b (mg/g) | 0.098 | 0.102 | 0.103 | 0.015 |
| DFAT (mg/g) | 0.321 | 0.319 | 0.320 | 0.045 |
| LK (%) | 27.53 | 26.96 | 28.58 | 4.65 |
| Cellulose (mg/g) | 428.9 | 443.3 | 400. 3 | 34.9 |
| Galacturonic acid (mg/g) | 8.939 | 8.907 | 8.852 | 2.366 |
| Glucuronic acid (mg/g) | 2.987 | 2.688 | 3.425 | 0.800 |
| Glucose (mg/g) | 40.13 | 38.83 | 32.99 | 8.609 |
| Galactose (mg/g) | 6.167 | 5.056 | 7.545 | 2.829 |
| Fucose (mg/g) | 7.593 | 6.115 | 6.949 | 2.341 |
| Arabinose (mg/g) | 9.638 | 8.503 | 11.49 | 2.931 |
| Rhamnose (mg/g) | 0.450 | 0.302 | 0.598 | 0.295 |
| Xylose (mg/g) | 23.60 | 23.33 | 23.89 | 3.01 |
| Mannose (mg/g) | 3.085 | 2.253 | 3.011 | 0.801 |
| Total Hemicellulose (mg/g) | 102.6 | 96.25 | 98.75 | 16.82 |
| Arabinose:Xylose Ratio (mg/g) | 0.534 | 0.485 | 0.720 | 0.342 |
| **Plant height (cm)** | | | | |
| PCA (mg/g) | 12.43 | 12.88 | 13.59 | 1.075 |
| FA (mg/g) | 3.097 | 2.893 | 3.107 | 0.439 |
| DFA 8-5-l (mg/g) | 0.059 | 0.049 | 0.059 | 0.0088 |
| DFA 5-5 (mg/g) | 0.084 | 0.080 | 0.081 | 0.012 |
| DFA 8-O-4 (mg/g) | 0.083 | 0.074 | 0.076 | 0.017 |
| DFA 8-5-b (mg/g) | 0.099 | 0.098 | 0.106 | 0.016 |
| DFAT (mg/g) | 0.325 | 0.301 | 0.322 | 0.047 |
| LK (%) | 28.34 | 27.89 | 26.34 | 4.89 |
| Cellulose (mg/g) | 425.37 | 439.78 | 418.01 | 1.98 |
| Galacturonic acid (mg/g) | 9.642 | 7.294 | 8.815 | 2.480 |
| Glucuronic acid (mg/g) | 3.203 | 2.837 | 2.798 | 0.835 |
| Glucose (mg/g) | 39.06 | 39.41 | 33.93 | 9.142 |
| Galactose (mg/g) | 7.293 | 4.549 | 5.426 | 2.974 |

| | | | | |
|---|---|---|---|---|
| Fucose (mg/g) | 7.873 | 5.834 | 5.893 | 2.444 |
| Arabinose (mg/g) | 10.94 | 7.867 | 9.144 | 3.082 |
| Rhamnose (mg/g) | 0.554 | 0.234 | 0.388 | 0.313 |
| Xylose (mg/g) | 24.06 | 22.23 | 23.75 | 3.186 |
| Mannose (mg/g) | 3.161 | 2.404 | 2.655 | 0.828 |
| Total Hemicellulose (mg/g) | 105.8 | 92.66 | 92.78 | 17.50 |
| Arabinose:Xylose Ratio (mg/g) | 0.655 | 0.541 | 0.451 | 0.365 |
| **Internode length (cm)** | | | | |
| PCA (mg/g) | 11.79 | 14.52 | 12.54 | 1.142 |
| FA (mg/g) | 3.023 | 3.133 | 3.050 | 0.501 |
| DFA 8-5-l (mg/g) | 0.058 | 0.062 | 0.056 | 0.010 |
| DFA 5-5 (mg/g) | 0.079 | 0.090 | 0.082 | 0.013 |
| DFA 8-O-4 (mg/g) | 0.078 | 0.073 | 0.081 | 0.019 |
| DFA 8-5-b (mg/g) | 0.092 | 0.099 | 0.104 | 0.018 |
| DFAT (mg/g) | 0.307 | 0.324 | 0.323 | 0.053 |
| LK (%) | 26.68 | 30.27 | 27.24 | 5.57 |
| S subunit (%) | 56.81 | 58.03 | 56.83 | 1.360 |
| S:G ratio | 1.414 | 1.467 | 1.400 | 0.074 |
| G subunit (%) | 40.23 | 39.71 | 40.84 | 1.216 |
| Cellulose (mg/g) | 434.13 | 430.49 | 422.60 | 42.75 |
| Galacturonic acid (mg/g) | 8.982 | 10.06 | 8.592 | 2.856 |
| Glucuronic acid (mg/g) | 2.408 | 3.848 | 2.963 | 0.937 |
| Glucose (mg/g) | 37.97 | 34.66 | 38.11 | 10.33 |
| Galactose (mg/g) | 5.727 | 8.799 | 5.613 | 3.310 |
| Fucose (mg/g) | 6.391 | 8.264 | 6.611 | 2.083 |
| Arabinose (mg/g) | 9.490 | 13.11 | 8.991 | 3.44 |
| Rhamnose (mg/g) | 0.477 | 0.693 | 0.362 | 0.355 |
| Xylose (mg/g) | 23.91 | 24.36 | 23.29 | 3.64 |
| Mannose (mg/g) | 2.388 | 3.430 | 2.837 | 0.939 |
| Total Hemicellulose (mg/g) | 97.74 | 107.2 | 97.37 | 20.10 |
| Arabinose:Xylose Ratio (mg/g) | 0.458 | 0.916 | 0.516 | 0.404 |
| **Internode diameter (mm)** | | | | |
| PCA (mg/g) | 12.43 | 12.72 | 12.99 | 1.083 |
| FA (mg/g) | 2.947 | 3.081 | 3.117 | 0.436 |
| DFA 8-5-l (mg/g) | 0.063 | 0.055 | 0.057 | 0.009 |
| DFA 5-5 (mg/g) | 0.088 | 0.080 | 0.082 | 0.011 |
| DFA 8-O-4 (mg/g) | 0.0812 | 0.075 | 0.085 | 0.017 |
| DFA 8-5-b (mg/g) | 0.105 | 0.097 | 0.105 | 0.016 |
| DFAT (mg/g) | 0.338 | 0.307 | 0.328 | 0.046 |
| LK (%) | 28.20 | 27.38 | 27.54 | 4.834 |
| S subunit (%) | 56.7 | 57.4 | 56.7 | 1.17 |
| S:G ratio | 1.39 | 1.43 | 1.39 | 0.06 |

| | | | | |
|---|---|---|---|---|
| G subunit (%) | 41.1 | 40.2 | 40.8 | 1.04 |
| H Subunit (%) | 2.26 | 2.48 | 2.50 | 0.40 |
| Cellulose (mg/g) | 422.29 | 413.86 | 453.01 | 36.26 |
| Galacturonic acid (mg/g) | 8.783 | 9.876 | 7.128 | 2.462 |
| Glucuronic acid (mg/g) | 3.074 | 2.925 | 3.082 | 0.851 |
| Glucose (mg/g) | 32.27 | 39.65 | 38.33 | 8.877 |
| Galactose (mg/g) | 5.479 | 5.893 | 7.255 | 2.993 |
| Fucose (mg/g) | 5.752 | 7.421 | 6.714 | 2.450 |
| Arabinose (mg/g) | 9.193 | 9.540 | 10.66 | 3.111 |
| Rhamnose (mg/g) | 0.415 | 0.411 | 0.506 | 0.315 |
| Xylose (mg/g) | 24.59 | 23.86 | 22.11 | 3.203 |
| Mannose (mg/g) | 3.128 | 2.822 | 2.635 | 0.843 |
| Total Hemicellulose (mg/g) | 92.69 | 102.39 | 98.42 | 17.51 |
| Arabinose:Xylose Ratio (mg/g) | 0.492 | 0.506 | 0.778 | 0.355 |
| **Rind puncture resistance (kg/section)** | | | | |
| FA (mg/g) | 0.309 | 0.307 | 0.309 | 0.429 |
| DFA 8-O-4 (mg/g) | 0.072 | 0.085 | 0.073 | 0.017 |
| LK (%) | 26.95 | 27.63 | 28.18 | 4.91 |
| S subunit (%) | 56.60 | 56.92 | 57.53 | 1.182 |
| Glucose (mg/g) | 41.80 | 36.25 | 36.21 | 8.962 |
| Fucose (mg/g) | 5.922 | 6.656 | 8.131 | 2.443 |
| Rhamnose (mg/g) | 0.333 | 0.360 | 0.670 | 0.310 |
| Total Hemicellulose (mg/g) | 93.42 | 96.77 | 108.1 | 17.46 |
| Ratio Arabinose:Xylose (mg/g) | 0.540 | 0.468 | 0.777 | 0.357 |

PCA: *p*-coumaric acid; FA: Ferulic acid; DFA 8-5-l: Diferulic acid 8-5-Linear; DFA 8- o-4: Diferulic acid 8-O-4; DFA 8-5: Diferulic acid 8-5; DFA8-5-b: Diferulic acid 8-5-Benzofuran; DFAT: Total diferulic acids; LK: Klason Lignin.

* some missing data for individual traits and inbreds could interfere in the final ratio calculations of the groups

Supplementary Table 1: Means of 20 inbred lines evaluated for agronomic and stem description traits.

| | Agronomic Traits | | Stem Description Traits | | | |
|---|---|---|---|---|---|---|
| **NOMVAR** | **Biomass Yield (Mg/ha)** | **Stem Lodging (%)** | **Plant Height (cm)** | **Internode Length (cm)** | **Internode Diameter (mm)** | **Rind puncture Resistance (kg/section)** |
| A509 | 1.8 | 5.6 | 125.4 | 15.3 | 14.9 | 2.1 |
| A632 | 6.0 | 1.0 | 171.9 | 15.1 | 14.1 | 2.6 |
| A654 | 3.3 | 0.0 | 111.9 | 15.1 | 15.1 | 2.5 |
| C103 | 5.8 | 2.0 | 180.6 | 16.6 | 16.5 | 4.0 |
| CO348 | 4.0 | 0.6 | 124.6 | 14.0 | 16.4 | 3.6 |
| CO384 | 5.2 | 0.0 | 163.3 | 14.2 | 16.3 | 3.0 |
| CO442 | 5.2 | 0.0 | 144.1 | 13.5 | 16.0 | 2.3 |
| CO444 | 3.4 | 0.0 | 138.1 | 13.3 | 13.0 | 2.5 |
| EC212 | 3.0 | 0.8 | 138.4 | 15.0 | 15.1 | 4.4 |
| EP105 | 3.6 | 0.6 | 155.8 | 15.3 | 15.6 | 2.6 |
| EP125 | 2.4 | 0.8 | 151.8 | 17.5 | 15.2 | 2.0 |
| EP17 | 5.5 | 0.0 | 166.7 | 14.3 | 17.3 | 3.3 |
| EP42 | 2.5 | 5.0 | 131.4 | 14.7 | 14.7 | 2.6 |
| EP47 | 5.3 | 1.1 | 172.8 | 18.0 | 16.8 | 2.6 |
| EP53 | 2.2 | 0.0 | 115.5 | 16.4 | 15.9 | 3.2 |
| EP86 | 1.7 | 1.1 | 143.7 | 15.4 | 14.8 | 2.4 |
| F473 | 2.4 | 0.8 | 128.2 | 13.9 | 14.0 | 2.9 |
| PB130 | 2.5 | 0.0 | 121.5 | 14.3 | 18.1 | 2.6 |
| W182B | 2.4 | 18.8 | 126.8 | 14.6 | 17.5 | 2.4 |
| W64A | 3.7 | 2.3 | 140.9 | 14.3 | 18.7 | 2.8 |
| LSD [a](P<0.05) | 2.7 | - | 14.1 | 1.15 | 1.99 | 0.73 |

[a]: traits with LSD value are significant at the 0.05 significant level, according to Fisher protected LSD method. A hyphen indicates non-significant differences (P>0.05)

Dear editor,

Please find enclosed the manuscript **"Maize Cell Wall Composition Impacts Structural Characteristics of the Stems and thereby Biomass Yield"** to consider for publication in the Journal of Agricultural and Food Chemistry

**CORRESPONDING AUTHOR:**

Ana López-Malvar

Facultad de Biología, Departamento de Biología Vegetal y Ciencias del Suelo, Universidad de Vigo, As Lagoas Marcosende, Vigo 36310, Spain. Agrobiología Ambiental, Calidad de Suelos y Plantas (UVIGO), Unidad Asociada a la MBG (CSIC)

E-mails: alopezmalvar@uvigo.es

**EXPLANATION OF MANUSCRIPT'S SIGNIFICANCE:**

Maize stems sustain leaves and reproductive structures, functionally support the transport of water and nutrients and it is involved in plant defence to stress, thereby impacting in economically important applications. Research on cell wall composition and its influence in basic and applied aspects of maize stem strength would be important steps in maize breeding and improvement. Overall, in the current study we evaluated agronomical stem description traits and biomass yield in a subset of maize inbred lines that showed variability for cell wall composition, being the main goal to identify cell wall components that can be used in applied breeding programs. In this representative material, a S-type lignin accompanied by increases in *p*-coumaric acid would be in detriment of biomass yield, plant height and rind puncture resistance, whereas, cell walls richer in cellulose and with lower proportion of hemicellulose, would be beneficial for stalk strength. These results

prove that cell wall composition clearly influences structural characteristics of the maize stems and thereby can be useful in order to improve maize biomass yield.
